# Differentiating Embryonic Stem Cells Pass through ‘Temporal Windows’ That Mark Responsiveness to Exogenous and Paracrine Mesendoderm Inducing Signals

**DOI:** 10.1371/journal.pone.0010706

**Published:** 2010-05-19

**Authors:** Steven A. Jackson, Jacqueline Schiesser, Edouard G. Stanley, Andrew G. Elefanty

**Affiliations:** Monash Immunology and Stem Cell Laboratories, Monash University, Clayton, Victoria, Australia; University of Calgary, Canada

## Abstract

**Background:**

Mesendoderm induction during embryonic stem cell (ESC) differentiation *in vitro* is stimulated by the Transforming Growth Factor and Wingless (Wnt) families of growth factors.

**Principal Findings:**

We identified the periods during which Bone Morphogenetic Protein (BMP) 4, Wnt3a or Activin A were able to induce expression of the mesendoderm marker, *Mixl1*, in differentiating mouse ESCs. BMP4 and Wnt3a were required between differentiation day (d) 1.5 and 3 to most effectively induce *Mixl1*, whilst Activin A induced *Mixl1* expression in ESC when added between d2 and d4, indicating a subtle difference in the requirement for Activin receptor signalling in this process. Stimulation of ESCs with these factors at earlier or later times resulted in little *Mixl1* induction, suggesting that the differentiating ESCs passed through ‘temporal windows’ in which they sequentially gained and lost competence to respond to each growth factor. Inhibition of either Activin or Wnt signalling blocked *Mixl1* induction by any of the three mesendoderm-inducing factors. Mixing experiments in which chimeric EBs were formed between growth factor-treated and untreated ESCs revealed that BMP, Activin and Wnt signalling all contributed to the propagation of paracrine mesendoderm inducing signals between adjacent cells. Finally, we demonstrated that the differentiating cells passed through ‘exit gates’ after which point they were no longer dependent on signalling from inducing molecules for *Mixl1* expression.

**Conclusions:**

These studies suggest that differentiating ESCs are directed by an interconnected network of growth factors similar to those present in early embryos and that the timing of growth factor activity is critical for mesendoderm induction.

## Introduction

The in vitro differentiation of embryonic stem cells (ESCs) represents an accessible system for analyzing parameters influencing the early stages of lineage specification and commitment. During differentiation, ESCs pass through a series of developmental milestones that mirror those traversed by cells within the embryo [1]–[3]. For example, gene-profiling experiments indicate that differentiating mouse ESCs sequentially express genes marking successive stages of embryonic development, including *Oct4* and *Sox2* (inner cell mass), *Fgf5* (epiblast) and *Brachyury, Mixl1* and *Gsc* (primitive streak) [2]. Following the expression of these genes, induction of markers representing differentiated cell types can be observed, such as *Pdx1* (foregut endoderm), *Nkx2-5* (cardiac mesoderm) and β*H1 globin* (yolk sac erythroid cells) [2]. Thus, parallels exist between the differentiation pathways used by ESCs in vitro and the developmental roadmap followed by cells during the early stages of embryogenesis [4].

Not only is there a correspondence between the developmental pathways followed by cells in vitro and in vivo, but there is a similar concordance between the factors that induce and pattern ESCs and the embryo during differentiation. For example, induction of the primitive streak, the structural harbinger of mesendoderm formation in the embryo, requires the activity of a number of secreted growth factors (reviewed in [5]). Specifically, embryos lacking BMP4, Wnt3, nodal or their receptors, display gastrulation and mesendoderm patterning defects [6]–[14]. Similarly, in vitro studies on ESCs indicate that stimulation by these ligands initiates mesendoderm formation, as evidenced by the expression of primitive streak markers *Brachyury*, *Mixl1* and *Goosecoid* [15]–[18]. Indeed, inhibitor studies have demonstrated that Wnt and Activin (nodal) signalling is absolutely required for this process, indicating that fundamental parallels exist between the differentiation of early embryonic cell types in vitro and in vivo [16], [18]–[20].

In this study we determined the periods within which BMP4, Wnt3a and Activin A induced mesendoderm in differentiating mouse ESCs. These factors acted during discrete ‘temporal windows’ to induce expression of a GFP reporter gene targeted to the locus of the primitive streak gene, *Mixl1*. We demonstrated that endogenously produced factors propagated paracrine mesendodermal inducing signals through embryoid bodies (EBs). Finally, we observed that the differentiating cells passed through ‘exit gates’ after which point they were no longer dependent on signalling from inducing molecules for *Mixl1* expression. Overall, our study suggests that growth factor regulatory loops similar to those present in early embryos also exist within EBs. The timing of growth factor activity is critical for the initiation of mesendoderm formation from ESCs and paracrine signalling contributes to mesendoderm development.

## Results

### Maximal mesendoderm inducing activity of BMP4, Activin A and Wnt3a occurs within discrete temporal windows

We utilised a genetically modified mESC line, *Mixl1* ^GFP/w^ [17], in which sequences encoding GFP were inserted into one allele of *Mixl1*, a gene whose expression is restricted to the mesendodermal precursors of the primitive streak [21], [22]. GFP acts as a surrogate marker for expression of Mixl1, and indicates the emergence of nascent mesoderm and endoderm from differentiating ESCs.

**Figure 1:**
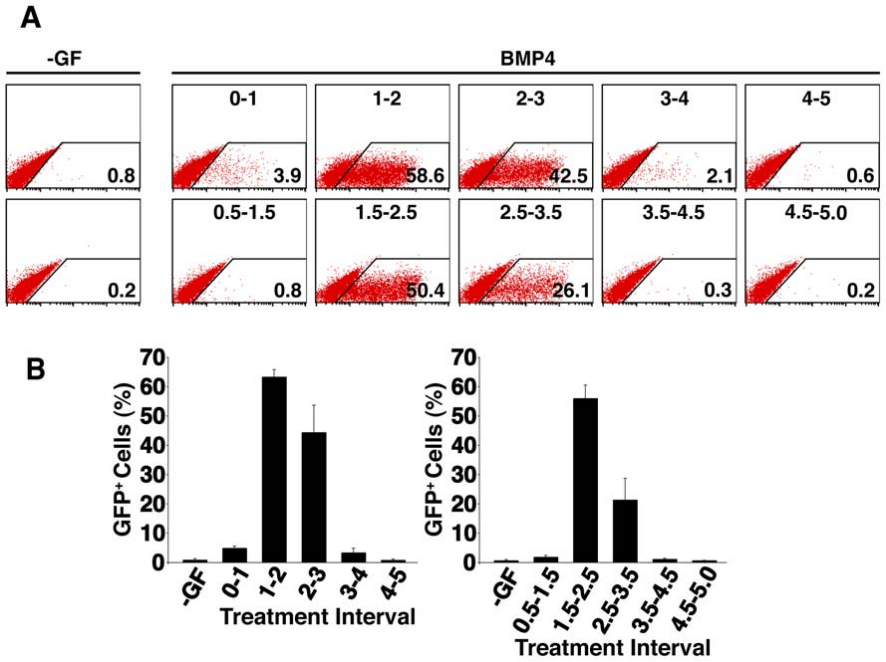
Mesendoderm inducing activity of BMP4 is restricted to a specific temporal window during ESC differentiation. (A) Flow cytometric analysis of d5 *Mixl1* ^GFP/w^ ESCs differentiated in cultures supplemented with 10 ng/ml BMP4 for 24 h with the time of initial growth factor addition to the culture staggered at daily intervals starting at d0 (upper panels) or day 0.5 (lower panels). The proportion of GFP^+^ cells in this experiment is shown in the lower right of each plot. Flow cytometry profiles from no growth factor (-GF) control cultures are shown to the left of each series. (B) Histograms summarising flow cytometry data from three independent experiments, showing the average percentage of GFP^+^ cells at d5 observed for each period of BMP4 addition. (mean±sd, *n = 3*).

**Figure 2:**
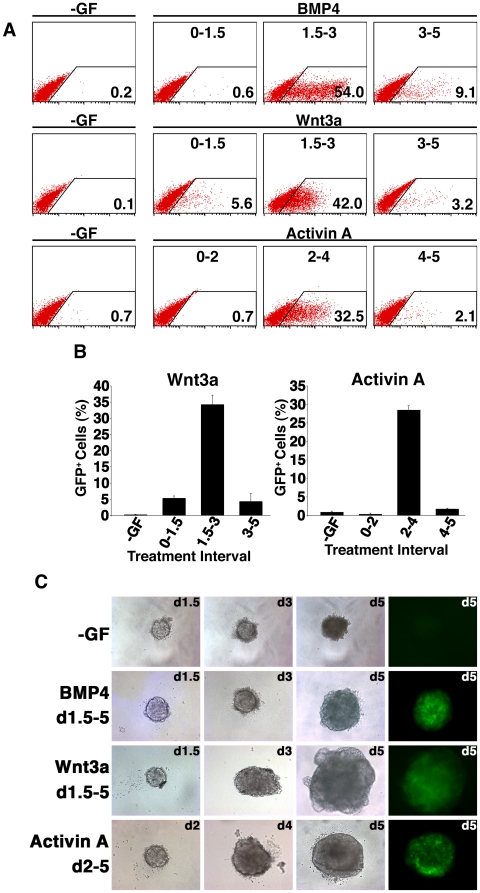
Mesendoderm inducing activities of BMP4, Wnt3a and Activin A are restricted to specific temporal windows during ESC differentiation. (A) Flow cytometry analysis at d5 of a representative experiment of *Mixl1* ^GFP/w^ ESCs differentiated in cultures supplemented with 10 ng/ml BMP4, 100 ng/ml Wnt3a or 100 ng/ml Activin A for the indicated time intervals (measured in days after initiation of differentiation). The proportion of GFP^+^ cells is shown in the lower right of each plot. The flow cytometry profiles from no growth factor (-GF) control cultures are shown to the left of each series. (B) Histogram summarising flow cytometry data measuring the proportion of GFP^+^ cells at d5 in EBs treated with Wnt3a or Activin A during the time intervals indicated (days) (mean±sd, *n = 3*). (C) Brightfield and epifluorescence images of differentiating EBs. The growth factors and period of addition are indicated to the left of each row and day of differentiation when the image was taken in the top right hand corner of each panel. (Original magnification×100).

In order to identify the period during differentiation when cells were responsive to mesendoderm inducing growth factors. *Mixl1* ^GFP/w^ ESCs were differentiated in a chemically defined medium (CDM) [23] supplemented with BMP4 for 24 h, with the time of initial addition to the culture staggered at daily intervals from d0 to d4 (Figure 1A). At the end of each 24 h period, the BMP4-supplemented medium was removed and the EBs left to differentiate further in fresh medium without growth factor. The cells were analysed for GFP expression by flow cytometry at d5, since the highest percentage of GFP^+^ cells were observed on this day, and expression rapidly waned thereafter. This was consistent with observations that GFP maturation and fluorescence lagged behind the peak of *Mixl1* mRNA expression that was maximal at d4 of differentiation [17], [24]. These experiments revealed that BMP4 most effectively induced expression of GFP from the *Mixl1* locus (denoted Mixl1GFP) when present in the cultures from d1–2 (63.2±2.6%; mean±sd of GFP^+^ cells from 3 independent experiments) and d2–3 (44.2±9.6%) (Figure 1B). Experiments in which the timing of BMP4 addition was offset by 12 h (Figure 1A) indicated that peak induction of Mixl1GFP^+^ cells was observed when BMP4 was added from d1.5–2.5 (55.8±4.6%). A lower frequency of GFP^+^ cells was seen in d5 cultures stimulated between d2.5 and d3.5 (21.2±7.4%) (Figure 1B). Finally, very few Mixl1GFP^+^ cells were induced by stimulating the cells from d0.5–1.5 or from d3–4. Combining these data sets suggested that cells would be maximally responsive to BMP4 between d1.5 and d3 of differentiation. This prediction was confirmed in the experiment shown in Figure 2A, in which over 85% of the cumulative total of GFP^+^ cells was observed in cultures stimulated with BMP4 between d1.5 and d3.

A similar series of 24 h pulse experiments conducted with Wnt3a and Activin A as the differentiation stimuli defined the optimal window for Wnt3a addition to be between d1.5 and d3 and that for Activin A to be between d2 and d4 (data not shown). On the basis of these preliminary studies, further differentiation experiments were performed in which the growth factors were included prior to, during and after the hypothesized optimal *Mixl1* induction window for each growth factor. These experiments confirmed that BMP4 and Wnt3a were most effective at inducing GFP^+^ mesendoderm precursors when present in the cultures between d1.5 and d3 (Figure 2A, B). In contrast, Activin A most efficiently induced GFP expression when present between d2 and d4 of differentiation (Figure 2A, B). The inclusion of each growth factor during its optimal window of action also increased the size and viability of the EBs compared to EBs cultured in CDM without growth factors (Figure 2C and data not shown). The larger EB size suggests that the growth factors probably influenced cell survival and proliferation as well as differentiation. Indeed, we have previously shown that supplementation of serum free medium with BMP4 augmented the total cell number and viability of differentiating mouse ESCs, and that this effect was observed prior to the induction of GFP in *Mixl1* ^GFP/w^ ES cells [17]. We have argued that this may correspond to the growth-promoting effect of BMP4 on epiblast cells prior to gastrulation [6], [11], [12].

Similar outcomes in experiments performed with an independently targeted *Mixl1* ^GFP/w^ reporter ESC line, (clone Mix 114) [17], indicated that the temporal windows delineated in this study for each growth factor were not specific for a single ESC line (Figure S1). Nevertheless, because the two *Mixl1* ^GFP/w^ clones were derived from the same parental ESC line, we cannot rule out the possibility that different strains of ESCs might vary in their propensity to differentiate. However, in our experience, ESC lines generally respond to growth factors in a similar manner, with the main differences being in the concentration of factors required for the development of specific cell types (Elizabeth Ng, EGS and AGE, unpublished results).

Even in our relatively well defined, short-term differentiation experiments, we observed that, at best, 60–70% of cells expressed GFP from the *Mixl1* locus, and that the variable fluorescence intensity observed indicated that not all Mixl1GFP expressing cells were identical. Interestingly, we have previously observed that this heterogeneity for Mixl1GFP expression is more evident within individual EBs than between EBs [17]. In other words, most EBs express Mixl1GFP but not all cells in each EB express GFP.

### Interdependence of signalling pathways in mesendoderm induction

We sought to determine whether all three growth factor signalling pathways were required for GFP induction in differentiating *Mixl1* ^GFP/w^ cells. In the first instance, we examined the effects of adding inhibitors for Wnt and TGF-beta pathways on the ability of each ligand to induce MixlGFP expressing cells. ESCs were treated with BMP4 or Wnt3a from d1 or with Activin A from d2, because preliminary experiments showed that addition of this factor prior to d2 inhibited ESC mesendoderm differentiation (data not shown). Inhibitors of BMP4- (noggin), Activin receptor- (SB 431542) or canonical Wnt- (Dkk-1) signalling were added to the cultures at the same time as the growth factors. Cultures were analysed at differentiation d5 for expression of GFP by flow cytometry.

**Figure 3:**
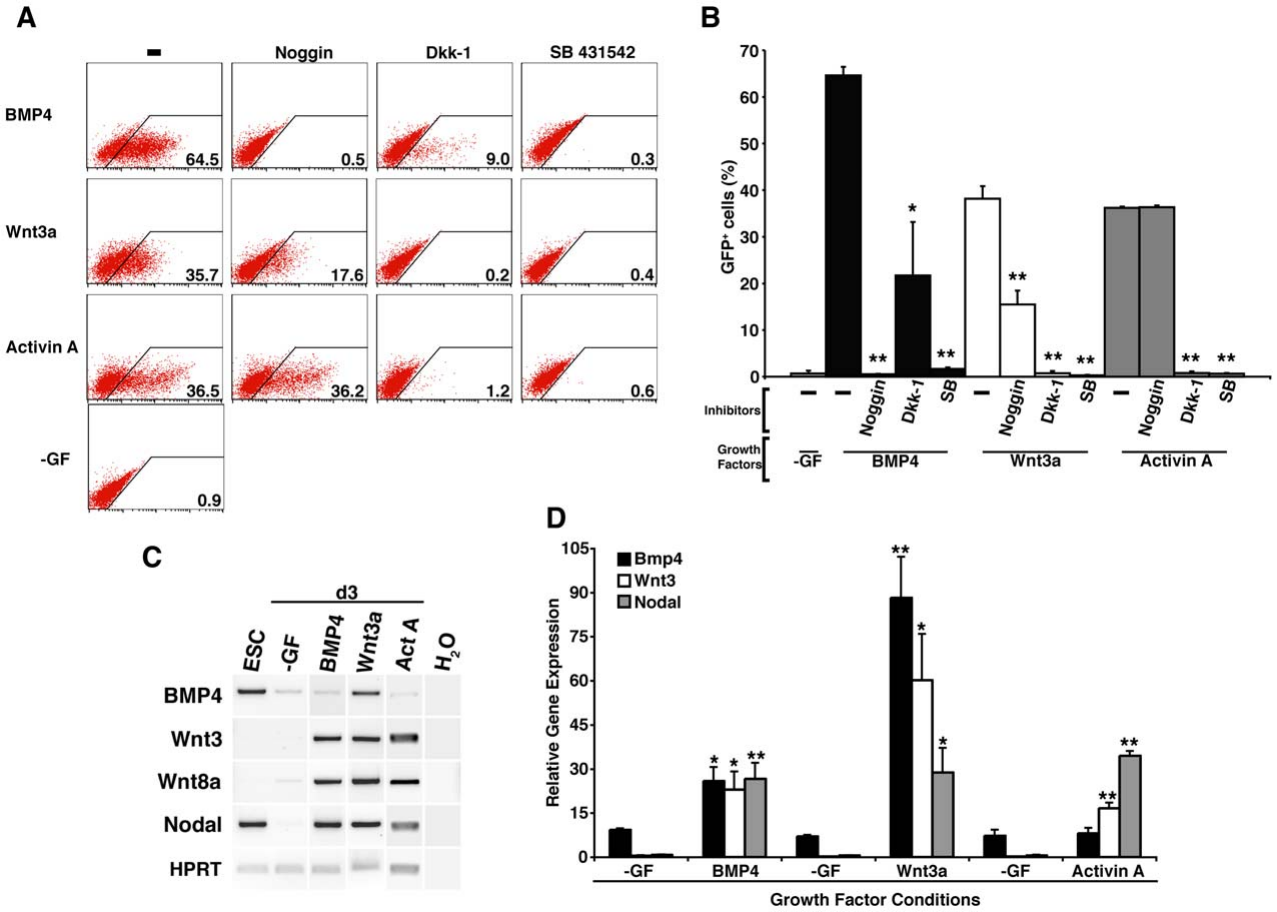
Induction of maximal proportions of GFP^+^ *Mixl1^GFP/w^* cells requires signalling via BMP, Wnt and Activin receptor pathways. (A) Flow cytometry analysis at d5 of a representative experiment of *Mixl1* ^GFP/w^ ESCs differentiated in cultures supplemented with BMP4 (d1–d5), Wnt3a (d1–d5) or Activin A (d2–d5) alone or in the presence of the signalling pathway inhibitors noggin, Dkk-1 or SB 431542. Growth factors are shown to the left of each row and inhibitors are shown at the top of each column. Flow cytometry profiles obtained from control cells with no growth factor added (-GF) are shown in the bottom left panel. The percentage of GFP^+^ positive cells is recorded in the bottom right corner of each plot. (B) Histogram summarising flow cytometry data measuring the proportion of GFP^+^ cells at d5 in EBs treated with BMP4, Wnt3a or Activin A with and without inhibitors. (mean±sd, *n = 3*) (* p<0.05, ** p<0.01 compared to cells not receiving inhibitor). SB; SB 431542. (C) Semi-quantitative RT-PCR analysis of growth factor gene expression in cells from d3 cultures stimulated with BMP4 (d1–d3), Wnt3a (d1–d3) or Activin A (d2–d3). The samples are indicated at the top of each column and the growth factor genes analysed on the left of each row. ESC; undifferentiated ESCs, -GF; no growth factor, Act A; Activin A, H_2_O; no template control. (D) Real time PCR analysis of *BMP4*, *Wnt3* and *nodal* gene expression at d3 in ESCs differentiated in the presence of BMP4 (d1.5–d3), Wnt3a (d1.5–d3) and Activin A (d2–d3). (mean±sem, *n = 3*). (* p<0.05, ** p<0.01 compared to samples collected from cells differentiated in the absence of growth factor.)

As anticipated, addition of a compound that inhibited the signalling pathway of the inducing agent completely ablated subsequent GFP-expression (Figure 3A). However, the Activin signalling inhibitor SB 431542 blocked GFP induction by all of the growth factors, underlining the pivotal role nodal signalling plays in mesendoderm formation [16], [18]. Somewhat unexpectedly, addition of noggin to Wnt3a treated cultures consistently suppressed induction of GFP-expressing cells by about 50% (Figure 3A, B), suggesting that endogenously produced BMP activity synergised with the exogenously added Wnt3a. In contrast, noggin had no impact on the frequency of GFP^+^ cells observed in d5 cultures which had been treated with Activin A (Figure 3A, B). The addition of Dkk-1 substantially reduced but did not eliminate subsequent induction of GFP^+^ cells in BMP4 treated cultures, and, in agreement with the results of others, *Mixl1* induction was obviated by Dkk-1 treatment of Activin A induced cultures (Figure 3A, B) [16].

In order to investigate the ligands endogenously produced by differentiating ESCs, PCR analysis was performed on cDNA synthesized from d3 EBs differentiated in the presence of either BMP4, Wnt3a or Activin A. This survey focused on genes encoding factors that previous gene profiling experiments had shown were expressed during ES differentiation [2]. BMP4 induced expression of *Wnt3*, *Wnt8a* and *nodal*, factors that may have contributed to endogenously produced signals inhibited by Dkk-1 or SB 431542 (Figure 3C). Likewise, d3 cells that had been treated with Wnt3a produced transcripts representing *BMP4*, *Wnt3*, *Wnt8a* and *nodal* (Figure 3C). The presence of *BMP4* transcripts in these samples may explain the suppressive effect of noggin on the frequency of Mixl1GFP^+^ cells induced by Wnt3a (Figure 3A, B). A similar analysis performed on d3 samples of Activin A treated cells revealed low levels of transcripts representing *Wnt3*, *Wnt8a* and *nodal*.

Real time PCR analysis confirmed the observation that both BMP4 and Wnt3a were able to induce substantial expression of *BMP4*, *Wnt3* and *nodal*. Activin A, only present for 24 hours at this time point, predominantly promoted the up regulation of *Wnt3* and *nodal*, with *BMP4* expression retained at similar levels to those observed in unstimulated cells (Figure 3D).

### Endogenously produced growth factors provide paracrine mesendoderm inducing signals in differentiating embryoid bodies

**Figure 4:**
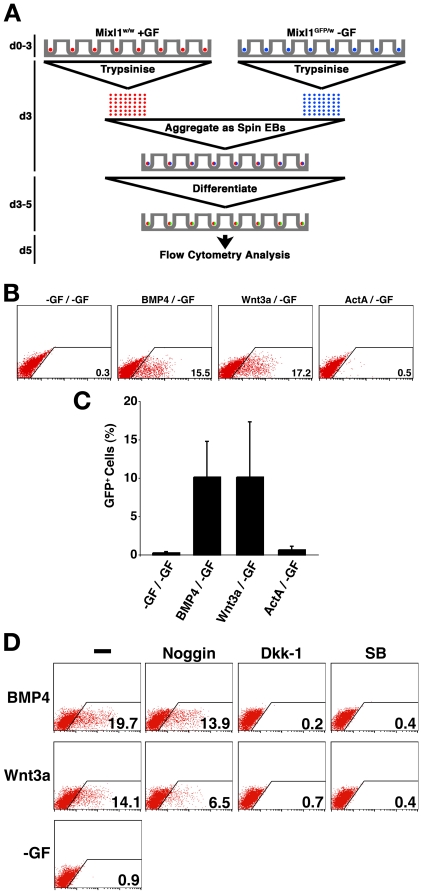
ES Cells differentiated in response to BMP4 or Wnt3a generate paracrine signals that induce GFP in *Mixl1* ^GFP/w^ EBs. (A) Method used to assess the ability of ‘stimulator’ wild type *Mixl1* ^w/w^ ESCs differentiated in the presence of growth factors to induce expression of GFP in ‘responder’ *Mixl1* ^GFP/w^ ESCs differentiated in absence of exogenous growth factors. After 3d of differentiation, both ‘stimulator’ and ‘responder’ EBs were disaggregated and chimeric spin EBs formed by re-aggregating ‘stimulator’ and ‘responder’ cells in a 1∶1 ratio. After allowing differentiation to proceed for a further 2d in the absence of growth factors the chimeric EBs were harvested for analysis. (B) Flow cytometry analysis of d5 chimeric EBs. The growth factors used for the ‘stimulator’ and ‘responder’ cultures for the initial 3d of differentiation are shown above each panel of a representative experiment (stimulator/responder). All ‘responder’ differentiations were performed in the absence of added growth factors (/-GF). The flow cytometry profiles obtained using ‘stimulator’ cells not exposed to growth factor (-GF/-GF) are shown as a negative control. The percentage of GFP^+^ cells is shown. (C) Histogram summarising the flow cytometry data at d5 (mean ±sd, *n = 3*). (D) Flow cytometry analysis of d5 chimeric EBs formed by aggregating growth factor stimulated wild type *Mixl1* ^w/w^ with unstimulated *Mixl1* ^GFP/w^ differentiating ESCs at d3. At the time of aggregation, inhibitors of BMP (noggin), canonical Wnt (Dkk-1) and Activin receptor (SB 431542) signalling were added to the cultures. Growth factors used to stimulate the wild type ESCs from d0–d3 are shown to the left of each row. Inhibitors are shown at the top of each column. Flow cytometry profiles obtained with cells from the no growth factor (-GF) control cultures are shown. The percentage of GFP^+^ positive cells is recorded in the bottom right corner of each plot. SB; SB 431542.

These results implied that endogenously produced BMP, Wnt and Activin-like molecules might play an important role during mesendoderm induction during ESC differentiation in vitro by complementing the actions of exogenously applied growth factors. To directly assay the paracrine mesendoderm inducing ability of these endogenous factors, we performed mixing experiments in which wild type *Mixl1* ^w/w^ ESCs differentiated for 3d in the presence of mesendoderm inducing factors were aggregated with *Mixl1* ^GFP/w^ cells that were derived from EBs differentiated for 3d in the absence of exogenous factors, to form chimeric EBs (Figure 4A). These chimeric spin EBs were left to differentiate for a further 2d in the absence of exogenous growth factors. We hypothesized that the wild type ‘stimulator’ EBs would differentiate towards mesendoderm in response to the growth factors during the first 3d but that the *Mixl1* ^GFP/w^ ‘responder’ EBs, cultured in the absence of stimulation, would not. Following the final 2d of differentiation as chimeric EBs, any mesendoderm inducing signal produced by the ‘stimulator’ cells transferred to the ‘responder’ *Mixl1* ^GFP/w^ cells would be read out as an induction of GFP^+^ cells by flow cytometry at d5. We chose the period of growth factor stimulation to include at least part of the optimal windows of response to BMP4, Wnt3a and Activin A. Furthermore, based on our earlier results, we argued that the period of chimeric EB differentiation (d3–d5) fell outside the window of optimal responsiveness of the EBs to direct BMP4 or Wnt3a induction of Mixl1GFP^+^ cells.

Analysis of Mixl1GFP expression in the chimeric EBs showed that, in the absence of exogenously added growth factors to the ‘stimulator’ cultures, no GFP^+^ cells were observed in the chimeric EBs at d5 (Figure 4B, C). Conversely, wild type *Mixl1* ^w/w^ ESCs differentiated for 3d in the presence of BMP4 or Wnt3a produced a mesendoderm-inducing signal that stimulated the ‘responder’ *Mixl1* ^GFP/w^ cells to induce GFP (Figure 4B, C). On average, 10.2±4.6% and 10.2±7.2% Mixl1GFP^+^ cells were observed in d5 chimeric EBs that included wild type cells stimulated from d0–d3 by BMP4 and Wnt3a respectively. Given that only ∼50% of the cells in each chimeric EB were ‘responder’ *Mixl1* ^GFP/w^ cells, these data argue that ∼20% of these cells upregulated *Mixl1* and expressed GFP in response to the co-cultivation with growth factor stimulated wild type ESCs. However, Activin A treated ‘stimulator’ wild type ESCs were not able to induce GFP expression in ‘responder’ *Mixl1* ^GFP/w^ cells (Figure 4B, C), perhaps reflecting the relatively low levels of growth factor gene expression observed in d3 EBs that had been stimulated with Activin A for only 24 hours (Figure 3C, D).

In order to dissect the requirements for BMP4, Wnt and Activin signaling pathways in the transfer of mesendoderm inducing signals, the effects of adding inhibitors of these pathways at the time of chimeric EB re-aggregation (at d3) was assessed. In the absence of inhibitors, wild type ‘stimulator’ ESCs treated with BMP4 or Wnt3a between d1–d3 effectively induced GFP expression in ‘responder’ *Mixl1* ^GFP/w^ cells (Figure 4D). Inclusion of Dkk-1 or SB 431542 completely abrogated transfer of the mesendoderm-inducing signal from the growth factor treated ‘stimulator’ cells to the *Mixl1* ^GFP/w^ ‘responder’ ESCs, indicating that signaling via these pathways was absolutely required (Figure 4D). In addition, noggin treatment of the chimeric EBs also diminished the frequency of GFP^+^ cells seen in the ‘responder’ *Mixl1* ^GFP/w^ ESCs (Figure 4D). This argued that BMP signalling still played a role in paracrine stimulation of mesendoderm formation, even though, by itself, BMP4 was a poor inducer of mesendoderm after d3. The failure of noggin to completely abrogate the induction of GFP expression confirmed that factors in addition to BMP4 mediated the paracrine signal transfer (compare Figure 4D with Figure 3A).

### BMP, Wnt and Activin signaling are required after d3 to maintain mesendoderm gene expression

Results of studies presented thus far suggested that the window during which BMP4 and Wnt3a efficiently induced GFP expression in *Mixl1* ^GFP/w^ cells closed soon after d3, consistent with the observation that addition of these growth factors after this time did not recruit many new cells into the mesendoderm differentiation program. However, this scenario did not exclude an ongoing requirement for active signaling past d3 for maximal GFP induction and/or maintenance in cells that had already committed to mesendoderm formation, a possibility raised by the effects of signaling pathway inhibitors on paracrine mesendoderm signals shown in Figure 4.

**Figure 5:**
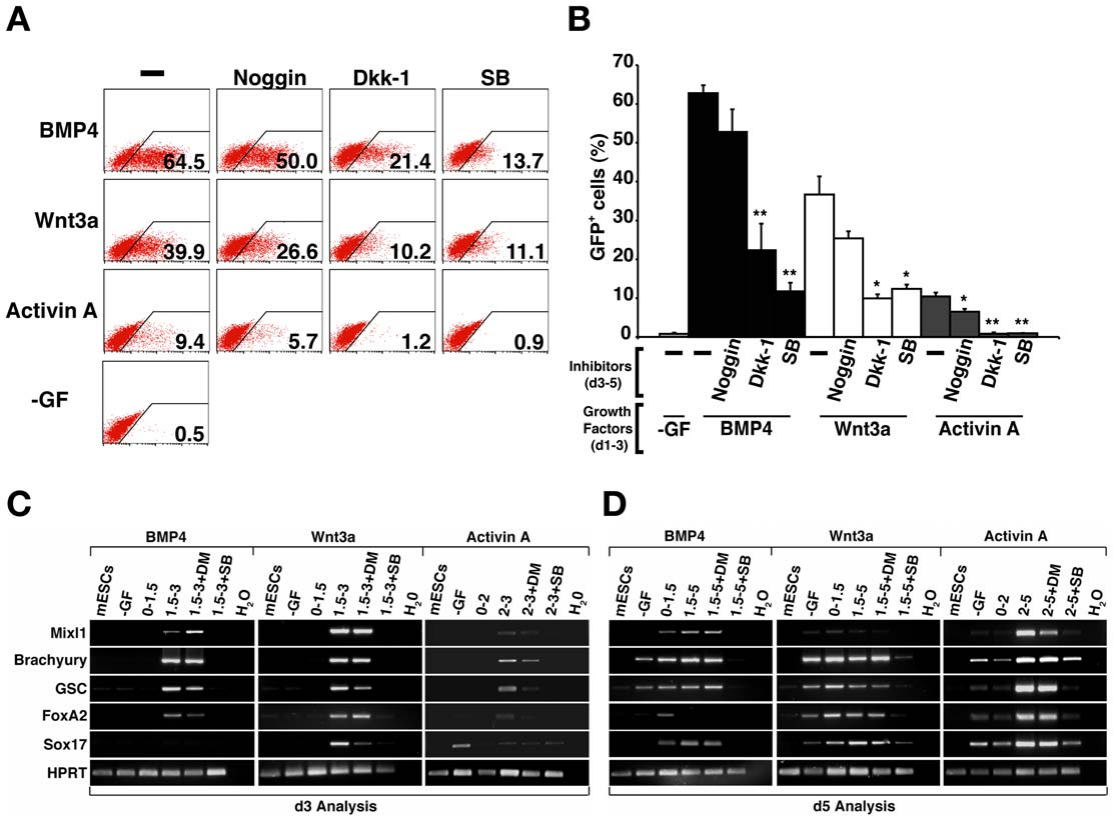
BMP4, Wnt and Activin signalling are required after d3 for maximal GFP induction from *Mixl1* ^GFP/w^ EBs. (A) Flow cytometry analysis of GFP expression in d5 *Mixl1* ^GFP/w^ EBs stimulated with BMP4 (d1–3), Wnt3a (d1–3) or Activin A (d2–3) and subsequently treated from d3–5 with the inhibitors noggin, Dkk-1 or SB 431542. SB; SB 431542. (B) Histogram summarising d5 flow cytometry data (mean±sd, *n = 3*) (* p<0.05, ** p<0.01 compared to cells not receiving inhibitor). SB; SB 431542. (C, D) Gene expression analysis of (C) d3 and (D) d5 differentiating mESCs treated with exogenous BMP4, Wnt3a and Activin A. The growth factor and treatment days are indicated across the top of each sample. Samples to which the Activin signalling inhibitor SB 431542 was added are indicated (SB), as are control samples that were treated with DMSO carrier (DM). The genes analysed are shown on the left hand side of each row.

Therefore, experiments were performed to examine the requirement for BMP, Wnt and Activin signaling after an initial period of mesendoderm induction by each growth factor. *Mixl1* ^GFP/w^ cells were differentiated until d3 in the presence of BMP4 or Wnt3a (both added at d1) or Activin A (added d2). At d3, the factors were removed and cells differentiated for a further two days in the presence or absence of inhibitors affecting each pathway (Figure 5A). Gene expression analysis indicated that by d3, cells treated with BMP4 and Wnt3a had up-regulated expression of the pan-mesendodermal markers, *Brachyury* and *Mixl1*, the anterior mesendodermal genes *Goosecoid* and *FoxA2* and the visceral and definitive endodermal marker *Sox17* (Figure 5C and real time PCR data shown in Figure S2). In the case of *Mixl1*, this expression at d3 translated into a substantial fraction of GFP^+^ cells by d5 (Figure 5A, B). However, much lower levels of *Mixl1* and *Brachyury* were expressed by d3 in response to Activin A, which was only present in these experiments for 24 h (Figure 5C and Figure S2). This correlated with the induction of GFP expression in only ∼10% of cells by d5, a proportion that represents only ∼30% of the d5 expression observed when Activin was included from d2–d4.

In all cases, inclusion of either noggin, Dkk-1 or SB 431542 at d3 reduced the proportion of GFP^+^ cells at d5 to varying degrees, suggesting that ongoing signaling through all of the pathways was required for maximal mesendoderm formation in response to each inducing growth factor. However, differences were observed in the patterns of GFP expression that largely depended upon the inhibitor that was used. In cells stimulated for 3d by BMP4, inclusion of noggin at d3 reduced the frequency of GFP^+^ cells by ∼20% (from 67.8±2.0% to 52.7±5.9%) whilst Dkk-1 and SB 431542 reduced the proportion of GFP expressing cells by ∼70% (from 67.8±2.0% to 22.3±6.9%) and ∼80% (from 67.8±2.0% to 11.7±2.3%) respectively (Fig. 5B). Similarly, in the case of cells stimulated by Wnt 3a or Activin A, the greatest reduction in the fraction of GFP^+^ cells was seen following addition of Dkk-1 and SB 431542 (∼75% and ∼90% respectively), whilst a lesser reduction in the proportion of GFP^+^ cells was observed in response to treatment of cells at d3 with noggin (∼35%) (Figure 5B). Interestingly, for cells stimulated by either BMP4 or Wnt3a, inclusion of the SB 431542 inhibitor at d3 only partially inhibited the appearance of GFP^+^ cells at d5 compared with results obtained when the inhibitor was included from the onset of the differentiation, which completely suppressed induction of Mixl1GFP^+^ cells (see Figure 3B). This indicated that a proportion of d5 Mixl1GFP^+^ cells were committed to mesendoderm formation by d3 and no longer dependent upon nodal signaling during the last 2 days of differentiation.

Examination of gene expression at d5 demonstrated that *Mixl1* RNA had begun to wane in cells stimulated by BMP4 or Wnt3a (Figure 5D). Conversely, d5 *Mixl1* expression was increased over d3 levels in Activin A stimulated cultures, illustrating differences in the kinetics of *Mixl1* induction. *Brachyury*, *Goosecoid*, *FoxA2* and *Sox17* were also expressed at higher levels in d5 Activin A stimulated samples. In all these cases, including the inhibitor SB 431542 significantly reduced gene expression, confirming that induction was dependent upon Activin A/nodal signaling. These trends in gene expression induced in response to BMP4, Wnt3a and Activin A stimulation were confirmed in an independent series of experiments (Figure S2).

**Figure 6:**
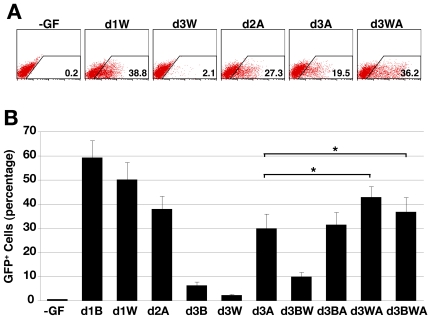
Wnt3a and Activin A synergise to induce GFP in *Mixl1* ^GFP/w^ EBs. (A) Flow cytometry analysis at d5 of a representative experiment of *Mixl1* ^GFP/w^ ESCs differentiated in cultures supplemented with 100 ng/ml Wnt3a (W) and/or 100 ng/ml Activin A (A) from the time indicated. The no growth factor (-GF) control is shown to the left. (B) Histogram summarising the d5 flow cytometry data from *Mixl1* ^GFP/w^ ESCs differentiated in cultures supplemented with the indicated growth factors from the time indicated. (mean±sd, *n = 3*) (* p<0.05 compared to d3A). B; BMP4, W; Wnt3a, A; Activin A.

The observation that Dkk-1 addition at d3 prevented the emergence of GFP^+^ cells in EBs stimulated with Activin A, suggested that endogenously produced Wnt ligands were necessary for Activin A to recruit cells to mesendoderm formation, even after the window for optimal Wnt3a induction of *Mixl1* expression appeared to have passed. A corollary of this hypothesis would be that Wnt3a might synergise with Activin A in the induction of mesendoderm after d3. To test this hypothesis, we analysed the induction of *Mixl1* in response to combinations of growth factors added to cells at d3. Consistent with our earlier results, these experiments confirmed that Wnt3a and BMP4 were poor inducers of GFP expression at d3, but that treatment of d3 cells with Activin A resulted in ∼30% GFP expressing cells at d5 (Figure 6A, B). Furthermore, there was no evidence for synergy between BMP4 and Wnt3a or BMP4 and Activin A added at d3 since the frequency of GFP^+^ cells was no higher than that observed by treating the cells with the single factors. However, addition of Wnt3a together with Activin A consistently resulted in a higher proportion of GFP^+^ cells (42.6±4.7%) than that seen with either ligand alone (2.0±0.7% for Wnt3a and 29.6±6.3% for Activin A) and higher than that predicted by addition of contributions representing the individual factors (Figure 6A, B). The inclusion of all three factors did not further increase the percentage of GFP expressing cells. These results suggest a second function for Wnt signaling as a necessary but not sufficient component in the late induction or maintenance of GFP^+^ mesendoderm distinct from its role as a direct inducer of early mesendoderm formation.

## Discussion

Before discussing the specific findings of this study in detail, it is valuable to place our results into a historical context, by relating our work to earlier studies that identified the nexus between ESCs and germ cell tumours. Benign germ cell tumours (teratomas) comprise mixtures of many different adult tissue types, whilst their malignant counterparts (teratocarcinomas) also include persistent undifferentiated stem cell components, termed embryonal carcinoma cells (ECCs) [25]. A number of excellent reviews over the years have covered this topic and the reader is referred to these for more complete descriptions of the research [25]–[28]. The concept that the multiple differentiated cell lineages found in teratomas might be derived from a single cell type was proposed over 100 years ago [29]. However, it was not until the 1950s, when Stevens and Little observed that inbred strain 129 mice developed spontaneous testicular teratomas, that there was an opportunity to systematically study these interesting tumours [30]. Stevens noted that teratocarcinomas maintained as an ascites tumour formed “thousands of free floating embryoid bodies similar to mouse embryos 5 and 6 days of age in the peritoneal fluid.” [31]. In a technical tour de force, Kleinsmith and Pierce dissociated small embryoid bodies (which contained a high proportion of undifferentiated ECCs) from an ascites tumour and transplanted single cells intraperitoneally, successfully generating clonal tumorigenic ECC lines [32]. The ability of these clonal tumours to differentiate into many different tissue types formally demonstrated the multipotentiality of the ECCs. This data was complemented by the demonstration that teratocarcinoma cells cultured on irradiated feeder cells could also be cloned in vitro and that these clones were also mulipotential [33]. Martin and Evans characterised in detail the in vitro culture and differentiation of ECCs [25], [26], [34]. They demonstrated that undifferentiated ECCs maintained on a mitotically inactivated feeder cell layer (later recognized as a source of the differentiation inhibiting factor, LIF[35]) would form embryoid bodies when cultured for a few days in suspension in serum containing medium, and that allowing the cystic embryoid bodies to reattach to the tissue culture dish triggered further differentiation to many different tissue types, an observation confirmed by others [25], [34], [36]. These scientists recorded two key observations that have been borne out over subsequent decades. Firstly, they observed that tissues formed from ECCs differentiated in vitro retained a degree of structural organisation reminiscent of normal embryonic development, and secondly they noted “that the processes of cell determination and differentiation occur in defined stages which are accessible to experimental analysis and manipulation.” [25], [34]. Indeed, Strickland and Mahdavi later showed that retinoic acid induced parietal endoderm differentiation from F9 ECCs [37].

The link between normal embryos and teratocarcinomas had been made when Solter [38] and Stevens [39] showed that transplantation of early mouse embryos to an extrauterine site led to the development of transplantable teratocarcinomas. The eventual derivation of ESCs, which phenotypically resembled ECCs, from preimplantation mouse blastocysts in 1981 independently by Evans and Martin [40], [41] shifted interest away from teratocarcinomas and ECCs and marked the beginning of the next era in pluripotent cell research, which has gained further momentum following the derivation of human embryonic stem cell lines in 1998 [42], [43] and the reprogramming of somatic cells to a pluripotent state reported in 2006 [44].

We have built on these earlier observations though our investigations of the induction of mesendoderm precursors by exogenously acting growth factors in differentiating mouse ESCs. Whilst early studies proved that ECCs (and later ESCs) could differentiate to form derivatives of the germ layers, the signals initiating differentiation were provided by serum and a specific dissection of the control mechanisms was not possible. We have used a suspension, embryoid body differentiation system, in which a serum free defined medium enabled us to objectively assess the influence of specific growth factors. Our studies were also aided by the use of a genetically modified ES cell line in which the induction of *Mixl1*, a homeobox gene that marks the primitive streak of the mammalian embryo, was linked to a fluorescent reporter [17], [21], [45]. Whilst numerous recent studies prior to ours have identified factors that induce and pattern mesoderm and endoderm (reviewed in [46]), we have defined temporal limits that constrain this process. We have shown that ESCs pass through a series of ‘windows’ in which they gain and lose competence to respond to three inducers of primitive streak transcription factors, BMP4, Wnt3a and Activin A. Through a series of mixing experiments, we demonstrated that endogenously generated TGF-beta - and Wnt-family growth factors induced by BMP4 and Wnt3a could propagate mesendoderm signals in differentiating EBs in a paracrine manner. Finally, we showed that a portion of the differentiating cells were committed to mesendoderm formation by d3 and did not require further signalling from inducing molecules during the last 2 days of differentiation for *Mixl1* expression.

Following the removal of the anti-differentiative signal, LIF, cells gained responsiveness to BMP4 and Wnt3a as mesendoderm inducing signals between d1.5 and d3, at a time corresponding to their upregulation of epiblast associated genes, such as *FGF5* [2]. This epiblast stage in ES cells, recently characterised by the (reversible) transition of cells from a Rex1^+^Oct4^+^ ESC phenotype to a Rex1^-^Oct4^+^ epiblast-like state [47], may be analogous to embryo derived pluripotent epiblast stem cells that are dependent on Activin and FGF signalling [48]. In this regard, our observations that Activin A treatment from day 0 to d2 maintained high levels of E-Cadherin and did not induce substantial GFP expression from *Mixl1* ^GFP/w^ cells (data not shown), were consistent with the hypothesized anti-differentiative role for nodal (which signals via the Activin receptor) during the earliest stages of differentiation [48]–[50]. Consistent with the hypothesis that the epiblast state correlates with BMP4 responsiveness, recent data demonstrates that differentiation of primordial germ cells from epiblast stem cells is a BMP4-dependent process [51].

From d3 of mESC differentiation, the window of competence began to close with cells no longer responding to BMP4 and Wnt signals alone, although cells remained Activin A responsive for a further day. This extended temporal window for mesendoderm induction by Activin A may reflect the prolonged role of nodal in maintaining the anterior streak at the latter stages of gastrulation [52], [53]. In fact, gene expression analysis indicated that whilst both BMP4 and Wnt3a robustly induced the pan-mesendoderm markers *Brachyury* and *Mixl1* at d3, they only weakly up-regulated the anterior mesendoderm/early endoderm markers *Goosecoid, Foxa2*, and *Sox17*. Conversely, Activin A induced higher levels of these genes at d5 of differentiation (Figure 5 and Figure S2). These data are in accordance with the results reported by others that BMP4 and Wnt3a signals induced predominantly a posterior primitive streak mesoderm in differentiating ESCs whilst Activin A biased differentiation towards anterior primitive streak derivatives including definitive endoderm [16], [18]–[20], [54].

**Figure 7:**
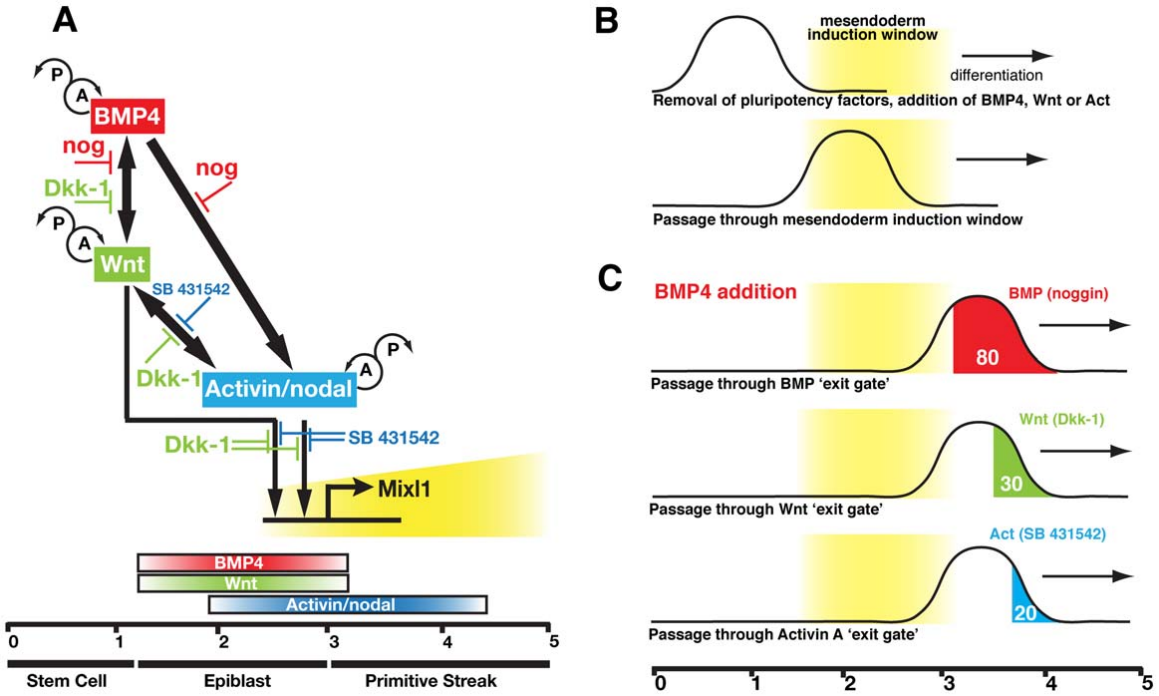
Induction of *Mixl1* expression is regulated through the integration of signals from BMP, Wnt and Activin/nodal pathways. (A) Interactions between signalling pathways and inhibitors impacting upon *Mixl1* induction. BMP4 stimulates expression of Wnt and Activin/nodal, which in turn induce *Mixl1,* perhaps acting through as yet unidentified intermediate molecules. The time periods (in days) and differentiation stages during which the differentiating ES cells are responsive to each stimulus are indicated. Probable autocrine (A) and paracrine (P) roles of the factors are indicated. (B) Removal of factors maintaining pluripotency enables ES cells to differentiate and to respond to BMP4, Wnt3a or Activin A signals delivered during a defined ‘temporal window’ for mesendoderm induction. (C) After cells pass through the mesendoderm window at d3, they then pass through ‘exit gates’ for each signalling pathway, after which time they are no longer dependent on that pathway for mesendoderm induction. In response to BMP4 addition between d1.5 and d3, the approximate percentage of cells that have passed the BMP4, Wnt3a or Activin A ‘exit gates’ at d3 is shown. See text for more details (Data taken from Figure 5B).

Our experiments illustrate the integration of signalling pathways required for induction of *Mixl1* (summarised in Figure 7A). In BMP4 stimulated cultures, transcription of *BMP4*, *Wnt3*, *Wnt8a* and *nodal* were induced and inhibition of either BMP or nodal signalling pathways eliminated Mixl1GFP expression. The consistent persistence of a residual percentage of Mixl1GFP^+^ cells in the presence of Dkk-1 argued that some BMP4 mediated mesendoderm differentiation might be Wnt independent. Induction of Mixl1GFP^+^ cells in Wnt3a or Activin A stimulated cultures was completely abrogated by inhibitors of either pathway. Treatment of Wnt3a-stimulated cultures with noggin consistently reduced the percentage of Mixl1GFP-expressing cells, perhaps suggesting a functional consequence of the significant level of *BMP4* transcription induced by Wnt3a. Conversely, treatment with noggin had little effect on Mixl1GFP induction by Activin A. These in vitro results contrast with findings in the embryo, in which a block in BMP4 signalling abrogates mesoderm induction because the embryo is then unable to produce downstream Wnt and nodal, a block that can be bypassed through the addition of exogenous factors in vitro. Our results are also in general agreement with knockout studies in the mouse [7]–[11], [14] and inhibitor experiments in ESCs [16], [18]–[20] that indicated that Wnt and Activin signalling were essential for mesendoderm induction.

Our data demonstrating that BMP4 propagates mesendoderm-inducing signals in differentiating EBs via the induction of endogenous TGF and Wnt growth factors are also consistent with the autoregulatory induction loops proposed to initiate and maintain gastrulation in mouse embryos [55]. In this model, uncleaved nodal protein first up-regulates expression of *BMP4* in the extraembryonic ectoderm. In turn, extraembryonic BMP4 then signals to the embryo proper to initiate *Wnt3* expression in the posterior visceral endoderm and epiblast. Wnt3 specifies mesoderm and acts to maintain *nodal* expression within the epiblast [55]. We explored this integration of signalling pathways through mixing experiments which showed that ESCs differentiated in response to BMP4 or Wnt3a propagated a paracrine GFP-inducing signal to d3 unstimulated *Mixl1* ^GFP/w^ EBs. Intriguingly, the transferred inductive signals were blocked not only by nodal inhibition, but also by Dkk-1 and partly by noggin, indicating a requirement for Wnt and BMP4 signalling even after closure of the window for response to these factors at d3. Studies in which these factors were added alone and in combination at d3 of differentiation confirmed our findings that direct BMP4 and Wnt3a responsiveness were greatly reduced after d3 but demonstrated that a significant population of cells were still responsive to Activin A. This observation suggests that *nodal* is a strong candidate for the predominant paracrine signalling molecule. The synergy that we observed between Activin and Wnt3a has previously been reported in normal mouse development [56] and in the context of human cancers [57], [58]. Mechanistically, in our experiments the results might reflect induction of *nodal* and its co receptor *cripto* (data not shown), by Wnt3a and Activin A. This synergy is also consistent with results reported by Hansson and colleagues who noted the late requirement for Wnt signalling in Activin A induction of Sox17^+^ definitive endoderm [20].

The higher frequency of d5 GFP^+^ cells observed in cultures where growth factors were removed at d3 compared with cultures that were also treated with inhibitors, suggested that mesendoderm formation remained dependent upon a growth factor for a short period even after it was removed from the culture. Experiments in which we evaluated the effects of adding inhibitors to growth factor induced cultures after d3 confirmed that the requirement for BMP4 was lost earlier than dependence upon Wnt or nodal signalling. This was evidenced by the higher percentages of d5 Mixl1GFP^+^ cells in noggin treated cultures compared to cultures in which Wnt or Activin signalling was inhibited (Figure 5B). In response to BMP4 stimulation, the frequency of GFP^+^ cells in d3 noggin treated cultures was ∼80% of the frequency without inhibitors whilst treatment with Dkk-1 or SB 431542 reduced the frequency of GFP^+^ cells to ∼30% and ∼20% of this value respectively. Similarly, in cultures stimulated by Wnt3a or Activin A, the frequency of GFP^+^ cells in d3 noggin treated cultures was ∼65% of the frequency without inhibitors whilst the inclusion of either Dkk-1 or SB 431542 reduced the frequency of GFP^+^ cells to ∼25% of the untreated value for Wnt stimulated cultures and ∼10% of this value for Activin A stimulated cultures. These data suggested that the wave of prospective mesendoderm passed through a series of ‘gates’ which marked its ‘exit’ from dependence on BMP4, Wnt and Activin/nodal signalling (Figure 7B). These ‘exit gates’ corresponded to the point at which addition of inhibitors no longer diminished the subsequent appearance of Mixl1GFP^+^ cells. As such, the lesser reduction in the percentage of GFP^+^ cells observed at d5 in cultures receiving noggin at d3 compared with cultures receiving Dkk-1 or SB 431542, indicated that dependence on BMP signalling was lost earlier than the requirement for Wnt or Activin/nodal signalling.

Overall, this study showed that differentiating mouse ESCs passed through specific ‘temporal windows’ in which cells gained and lost responsiveness to particular factors. It also demonstrated the requirement for an integrated network of signalling molecules to maintain the process of mesendoderm induction. Further studies investigating the detailed molecular mechanisms underpinning these observations will provide additional insights into the regulation of the germ layers during mammalian development.

## Materials and Methods

### ESC growth and differentiation

The *Mixl1* ^w/w^ (W9.5) [59] and *Mixl1* ^GFP/w^ (clone Mix147 and Mix114) [17], [22] ESC lines were maintained as described [60]. Differentiation was initiated by allowing EBs to form in cultures of disaggregated ESCs seeded at 1×10^4^ cells/ml in non-adherent 6 cm bacteriological dishes (Phoenix Biomedical) or by spin EB formation with 5×10^2^ cells seeded per well in non-adherent round bottomed 96 well plates (Nunc) in a chemically defined medium (CDM) [17], [23] as described [61]. The growth factors BMP4 (10 ng/ml), Activin A (100 ng/ml) (both from R&D Systems) and Wnt3a (100 ng/ml) (Millipore) were added at the indicated times in each experiment. The effective concentrations of the nodal signalling inhibitor, SB 431542 (Sigma Aldrich), the BMP signalling inhibitor, noggin (R&D Systems) and the Wnt inhibitor, Dkk-1 (R & D Systems) were determined by titrating each factor into mESC cultures differentiated in the presence of 100 ng/ml Activin A, 10 ng/ml BMP4 and 100 ng/ml Wnt3a, respectively. These experiments showed that 4 µM SB 431542, 100 ng/ml noggin or 200 ng/ml Dkk-1 was sufficient to block ligand-induced GFP expression in *Mixl1* ^GFP/w^ cells. Growth factors were removed from the cultures by pelleting EBs by centrifugation (480×*g*), aspirating the media, washing once with PBS, and resuspending the EBs in fresh CDM. The EBs were then transferred to a non-adherent bacteriological dish and returned to a humidified 37°C incubator (8% CO_2_ in air). GFP expression was analysed by flow cytometry at day 5 of differentiation unless otherwise stated.

### Differentiation of chimeric embryoid bodies

*Mixl1* ^w/w^ and *Mixl1* ^GFP/w^ cells were differentiated as EBs in parallel cultures for 3d. *Mixl1* ^w/w^ EBs were differentiated in the absence of growth factors (control cells) or in the presence of BMP4 (10 ng/ml), Wnt3a (100 ng/ml) or Activin A (100 ng/ml) (stimulator cells). BMP4 and Wnt3a were included from the onset of differentiation whilst Activin A was added at d2. For the first 3d, *Mixl1* ^GFP/w^ cells (responder cells) were differentiated in the absence of added growth factors. After 3d, EBs from both lines were harvested, washed in PBS, and trypsinised to form a single cell suspension. The disaggregated *Mixl1* ^w/w^ and *Mixl1* ^GFP/w^ cells were combined at a ratio of 1∶1 in CDM. Two thousand cells were placed into each well of low adherent round bottomed 96 well trays (Nunc) and chimeric spin EBs were formed by aggregation of the cells following centrifugation [61]. Inhibitors of signalling were added as indicated. The differentiation was allowed to proceed until d5 when the chimeric EBs were disaggregated with trypsin and the cells were analysed by flow cytometry for GFP expression.

### Gene expression analysis

RNA was extracted using an RNeasy Mini Kit (Qiagen) and cDNA synthesized using SuperScript III (Invitrogen Corporation) according to the manufacturer's instructions. PCR gene expression analysis was performed as described previously [62]. Samples from separate experiments involving BMP4, Wnt3a and Activin A treatments were all standardised against the HPRT expression from a single RNA sample derived from undifferentiated mESCs. PCRs were carried out using Platinum Taq DNA Polymerase High Fidelity (Invitrogen) with the primer pairs and PCR conditions listed in Table S1. Quantitative real time Taqman gene expression analysis was performed according to the manufacturer's instructions using the probe sets listed in Table S2 and processed as described [63].

## Supporting Information

Figure S1 BMP4, Wnt3a and Activin A display a similar pattern of mesendoderm inducing activity in differentiating Mixl1GFP/w clone Mix 114. (A) Flow cytometry analysis of d5 Mixl1GFP/w clone 114 ESCs differentiated in cultures supplemented with 10ng/ml BMP4, 100ng/ml Wnt3a or 100ng/ml Activin A for the given time intervals, indicating the proportion of GFP+ cells. The growth factor treatment for each experiment is indicated and the corresponding no growth factor (-GF) control flow cytometry profiles are shown to the left of each series. (B) Representative brightfield and epifluorescence images of differentiating EBs. The growth factors and period of addition are indicated to the left of each row and day of differentiation when the image was taken in the top right hand corner of each panel. (Original magnification x 100).
(12.87 MB TIF)

Figure S2 Kinetics of mesendoderm gene expression in EBs differentiated in BMP4, Wnt3a and Activin A. Real time PCR analysis for the indicated genes at (A) d3 and (B) d5 in ESCs differentiated in the absence of growth factors (-GF) or presence of BMP4, Wnt3a or Activin A for the indicated periods (mean±sem, n = 3).
(2.19 MB TIF)

Table S1 Sequences of primers used for PCR analysis shown in Figure 2 and 5.
(0.04 MB DOC)

Table S2 Probe sets for real-time PCR analysis shown in Figure 3 and Figure S2.
(0.03 MB DOC)
