# Supplementary figures and images for: Differentiating Embryonic Stem Cells Pass through ‘Temporal Windows’ That Mark Responsiveness to Exogenous and Paracrine Mesendoderm Inducing Signals
Source: PLoS One. 2010 May 19;5(5):e10706. doi: 10.1371/journal.pone.0010706 (PMC2873409; doi:10.1371/journal.pone.0010706)

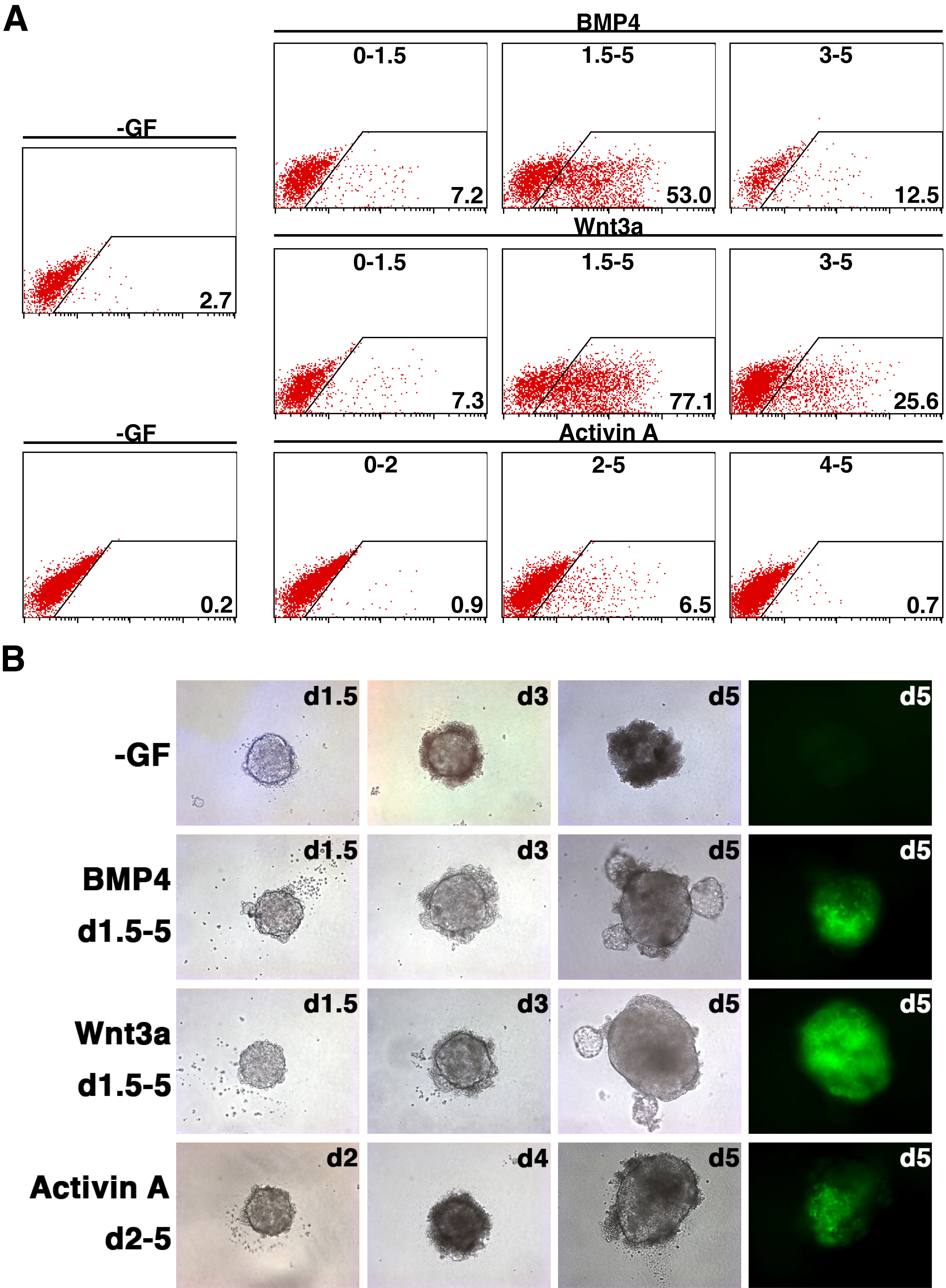

Supplement: Figure S1 — BMP4, Wnt3a and Activin A display a similar pattern of mesendoderm inducing activity in differentiating Mixl1GFP/w clone Mix 114. (A) Flow cytometry analysis of d5 Mixl1GFP/w clone 114 ESCs differentiated in cultures supplemented with 10ng/ml BMP4, 100ng/ml Wnt3a or 100ng/ml Activin A for the given time intervals, indicating the proportion of GFP+ cells. The growth factor treatment for each experiment is indicated and the corresponding no growth factor (-GF) control flow cytometry profiles are shown to the left of each series. (B) Representative brightfield and epifluorescence images of differentiating EBs. The growth factors and period of addition are indicated to the left of each row and day of differentiation when the image was taken in the top right hand corner of each panel. (Original magnification x 100). (12.87 MB TIF) [file pone.0010706.s001.tif]

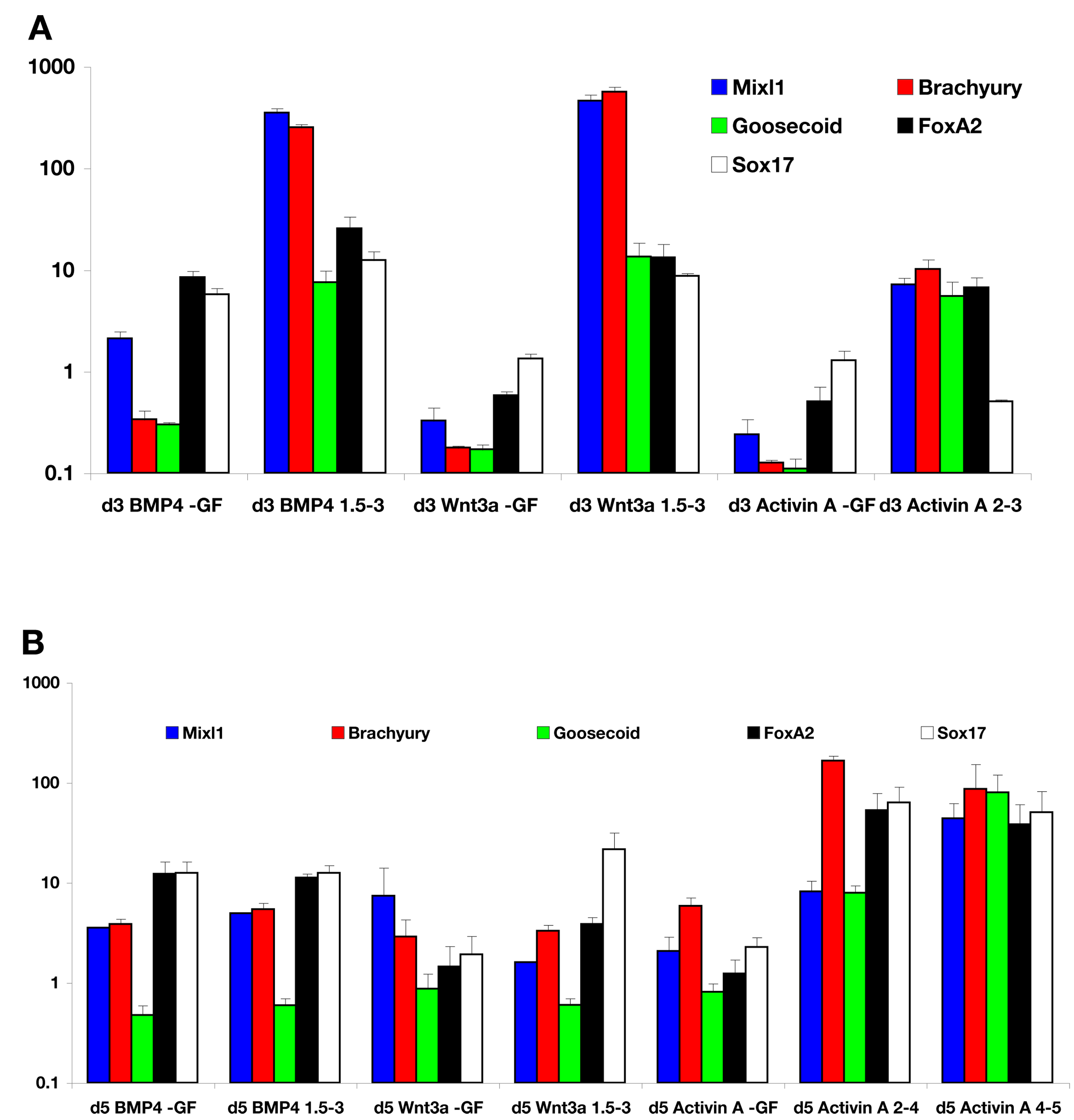

Supplement: Figure S2 — Kinetics of mesendoderm gene expression in EBs differentiated in BMP4, Wnt3a and Activin A. Real time PCR analysis for the indicated genes at (A) d3 and (B) d5 in ESCs differentiated in the absence of growth factors (-GF) or presence of BMP4, Wnt3a or Activin A for the indicated periods (mean±sem, n = 3). (2.19 MB TIF) [file pone.0010706.s002.tif]
