# Supplementary material for: Differentiating Embryonic Stem Cells Pass through ‘Temporal Windows’ That Mark Responsiveness to Exogenous and Paracrine Mesendoderm Inducing Signals
Source: PLoS One. 2010 May 19;5(5):e10706. doi: 10.1371/journal.pone.0010706 (PMC2873409; doi:10.1371/journal.pone.0010706)
Supplement: Table S1 — Sequences of primers used for PCR analysis shown in Figure 2 and 5. (0.04 MB DOC) [file pone.0010706.s003.doc]

**Table S1. Sequences of primers used for PCR analysis shown in Figure 2 and 5.**

| Gene | **5’ Primer** | **3’ Primer** | **Tm**  **(°C)** | **Size**  **(bp)** |
| --- | --- | --- | --- | --- |
| Mixl1 | GCACGTCGTTCAGCTCGGAGCAGC | AGTCATGCTGGGATCCGGAACGTGG | 60 | 320 |
| *Brachyury* | TGCTGCCTGTGAGTCATAAC | TCCAGGTGCTATATATTGCC | 55 | 948 |
| *Goosecoid* | GAGCAGCTGGCCAGGAAGGTGCAC | CAGCTAGCTCCTCGTTGCTTTCTC | 60 | 324 |
| *BMP4* | CAAACGTAGTCCCAAGCATCACCCAC | TCCGCCCTCTGGACTGCCTGATCTC | 60 | 381 |
| *Wnt3* | GGGTCTGCTAATGCTGGCTTGACGAG | CCTCCCATTGGATGCTATTACCTGAC | 60 | 407 |
| *Wnt8a* | GAGGCTGCAGCGACAACGTGGAGTTC | CCACACTTGACAGTGCAACACCACTG | 60 | 534 |
| *Nodal* | GGGACGCGTTTGCCAGACAGAAGCCAACTGTG | TCAGAGGCACCCACACTCCTCCAC | 60 | 336 |
| *FoxA2* | CCTCTATGTAGACTACTGCTTCTC | CCTGGATTTCACCATGTCCAGAATG | 60 | 227 |
| *Sox17* | TAACTCGCTGTTACAGTGCTTGGC | ATAGTAGACCGCTGAGCTAGCG | 60 | 489 |
| *HPRT* | GCTGGTGAAAAGGACCTCT | CACAGGACTAGAACACCTGC | 55 | 249 |
