# Supplementary material for: Differentiating Embryonic Stem Cells Pass through ‘Temporal Windows’ That Mark Responsiveness to Exogenous and Paracrine Mesendoderm Inducing Signals
Source: PLoS One. 2010 May 19;5(5):e10706. doi: 10.1371/journal.pone.0010706 (PMC2873409; doi:10.1371/journal.pone.0010706)
Supplement: Table S2 — Probe sets for real-time PCR analysis shown in Figure 3 and Figure S2. (0.03 MB DOC) [file pone.0010706.s004.doc]

**Table S2. Probe sets for real-time PCR analysis shown in Figure 3 and Figure S2.**

| Gene | **Probe Set** |
| --- | --- |
| *BMP4* | Mm00432087_m1 |
| *Nodal* | Mm03024078_m1 |
| *Wnt3* | Mm00437336_m1 |
| *Mixl1* | Mm00489085_m1 |
| *Brachyury* | Mm00436877_m1 |
| *Goosecoid* | Mm00650681_g1 |
| *Sox17* | Mm00488363_m1 |
| *FoxA2* | Mm00839704_mH |
| *Gapdh* | Mm99999915_g1 |
